# Folate-modified liposomal celastrol for rheumatoid arthritis: therapeutic efficacy and modulation of ROS/NF-κB/COX-2-related inflammatory signaling

**DOI:** 10.3389/fimmu.2026.1841496

**Published:** 2026-09-01

**Authors:** Jiquan Shen, Changjian Zhou, Xinggao Wang, Yuxuan Chen, Run Liu, Liang Hong, Weiping Ji, Bo Wang

**Affiliations:** 1 Department of Orthopedics, Lishui Hospital of Wenzhou Medical University, The First Affiliated Hospital of Lishui University, Lishui People’s Hospital, Lishui, Zhejiang, China; 2 Department of Orthopaedics, The People’s Hospital of Yunhe, Lishui, Zhejiang, China

**Keywords:** celastrol, folate-modified liposomes, rheumatoid arthritis, ROS/NF-κB/COX-2-related signaling, targeted delivery

## Abstract

**Background:**

Rheumatoid arthritis (RA) is a chronic autoimmune disorder characterized by severe synovial inflammation, joint deformities, and progressive tissue destruction. Celastrol (Cel), a natural triterpenoid with anti-inflammatory and antioxidant activity, is a promising therapeutic candidate for RA; however, its application is limited by poor aqueous solubility, low bioavailability, and systemic toxicity.

**Purpose:**

This study aimed to develop folate-modified liposomes encapsulating Cel (FA-Cel-LPs) to improve its solubility, stability, and joint accumulation, and to evaluate their anti-inflammatory efficacy and associated molecular changes in LPS-activated RAW264.7 macrophages and collagen-induced arthritis (CIA) rats.

**Methods:**

FA-Cel-LPs were prepared by thin-film hydration followed by extrusion and characterized for particle size, morphology, polydispersity index (PDI), encapsulation efficiency (EE), and *in vitro* release behavior. In LPS-activated RAW264.7 cells, cellular uptake, intracellular ROS levels, NF-κB nuclear translocation, COX-2/iNOS expression, and cytokine secretion were evaluated. In CIA rats, therapeutic efficacy was assessed by paw swelling, arthritis scores, *in vivo* fluorescence imaging, MRI, histological analysis, and micro-CT-based bone parameters. Safety was evaluated using hemolysis, serum biochemical analysis, and organ histology.

**Results:**

FA-Cel-LPs showed a mean particle size of 110.65 ± 1.24 nm, an EE of 84.67 ± 2.55%, a PDI of 0.230 ± 0.018, and a sustained release profile with pH-dependent differences *in vitro*. In RAW264.7 cells, folate-modified liposomes showed greater cellular uptake and were associated with reduced ROS levels, attenuated NF-κB activation, and decreased COX-2, iNOS, and pro-inflammatory cytokine expression, with low cytotoxicity. In CIA rats, *in vivo* imaging suggested greater accumulation of FA-Cel-LPs in inflamed joints. Compared with free Cel and Cel-LPs, FA-Cel-LPs more effectively reduced paw swelling and joint inflammation while attenuating bone erosion and preserving joint architecture. Serum ALT and AST levels in the FA-Cel-LPs group remained within physiological ranges and were significantly lower than those in the free Cel group. Organ histology showed no obvious treatment-related injury, suggesting acceptable preliminary safety.

**Conclusion:**

FA-Cel-LPs improved the solubility, formulation stability, and apparent joint accumulation of Cel and enhanced its anti-inflammatory and bone-protective effects in experimental RA. These effects were associated with reduced ROS/NF-κB/COX-2-related inflammatory changes, suggesting FA-Cel-LPs may represent a promising liposomal strategy for RA.

## 1 Introduction

Rheumatoid arthritis (RA) is a chronic systemic autoimmune disease characterized by persistent, non-infectious synovial inflammation and hyperplasia, typically affecting symmetric small joints (1, 2). Progressive synovitis leads to pain, swelling, stiffness, and functional impairment, ultimately resulting in severe joint deformity. Affecting approximately 0.3%–1% of the global population, RA imposes a substantial socioeconomic burden (3). Compared with individuals without RA, patients incur higher direct medical expenditures, with estimates of roughly USD 2,000 additional annual costs per patient (4). Over the past three decades, the incidence of RA has risen by approximately 8.2%, highlighting it as a growing global health challenge (5). Current therapeutic strategies primarily rely on nonsteroidal anti-inflammatory drugs (NSAIDs), glucocorticoids, disease-modifying antirheumatic drugs (DMARDs), and biologic agents such as tumor necrosis factor-alpha (TNF-α) inhibitors (6, 7). While these interventions can alleviate symptoms and decelerate disease progression, they often fail to achieve durable remission and are associated with significant adverse effects following prolonged use (8). Consequently, developing highly efficacious therapeutic approaches with improved safety profiles remains a critical priority in RA research.

Natural products derived from herbal medicine have emerged as promising candidates for RA management (9). *Tripterygium wilfordii Hook F.* (TwHF), a traditional Chinese medicinal herb used for over half a century in RA treatment, has garnered significant attention for its potent therapeutic efficacy (10). Celastrol (Cel), a major bioactive triterpenoid extracted from TwHF, is recognized as a potent immunomodulator with substantial potential in managing chronic autoimmune diseases (11–13). Previous studies have shown that Cel attenuates RA progression by regulating multiple signaling pathways (14–17). For instance, it inhibits macrophage polarization toward the pro-inflammatory M1 phenotype by suppressing the NF-κB and Notch1 pathways, thereby reducing pro-inflammatory cytokine production (16). Furthermore, Cel induces DNA damage, cell cycle arrest, and apoptosis in fibroblast-like synoviocytes (FLS), preventing abnormal synovial proliferation (18). Recent studies also indicate that Cel alleviates RA symptoms by targeting the ROS/NF-κB/NLRP3 signaling axis (15). However, the clinical translation of Cel is severely restricted by its poor aqueous solubility, short half-life, low bioavailability, narrow therapeutic window, and dose-dependent hepatorenal toxicity (19, 20). Accordingly, advanced nanoscale drug delivery systems (DDS) may provide a practical strategy for improving the pharmaceutical performance and therapeutic utility of Cel.

Liposomes are spherical vesicles composed of phospholipid bilayers and are widely used as biocompatible and biodegradable carriers for the encapsulation of both hydrophilic and hydrophobic drugs (21, 22). Surface modification with polyethylene glycol (PEG) further enhances their colloidal stability and prolongs *in vivo* circulation by evading the mononuclear phagocyte system (23). Previous studies have utilized PEGylated Cel-loaded liposomes, improving the bioavailability and anti-inflammatory effects of Cel in collagen-induced arthritis (CIA) models (24). Nevertheless, this passive delivery approach relies heavily on the enhanced permeability and retention (EPR) effect, which may be insufficient to prevent off-target biodistribution or hepatic clearance. To minimize systemic toxicity and maximize drug accumulation at the inflammatory site, active targeting strategies are urgently required (25, 26).

Folate (FA) receptor-mediated targeted drug delivery represents a highly efficient strategy for RA therapy. Crucially, activated macrophages, the primary drivers of RA synovial inflammation, overexpress folate receptor beta (FR-β), whereas folate receptor expression remains relatively low in most normal tissues. FA conjugation to the liposomal surface not only maintains prolonged circulation but also facilitates selective drug accumulation and internalization in inflamed joints via FR-β-mediated endocytosis (27, 28). For example, Wang et al. demonstrated that FA-modified micelles exhibited specific affinity toward RAW264.7 macrophages, effectively suppressing inflammatory cytokine secretion (29). Similarly, Maarten et al. highlighted the clinical potential of FA-modified PET tracers for macrophage imaging in RA patients, underscoring their promising applications in macrophage-driven chronic inflammatory diseases (30). On this basis, incorporation of Cel into FA-functionalized liposomes may provide dual advantages, namely improved solubility and enhanced delivery to activated macrophages within inflamed joints.

Although both PEGylated Cel-loaded liposomes and folate-modified nanocarriers have been reported previously, these approaches have often been investigated separately, and direct comparisons between non-targeted and FA-targeted Cel liposomal systems remain limited (31–33). Moreover, therapeutic evaluation has frequently focused on relatively macroscopic outcomes, such as paw swelling and routine histopathological assessment, whereas the incremental value of FA functionalization in terms of joint accumulation, bone protection, and preliminary systemic safety has not been fully characterized. Accordingly, the present study was designed not to establish an entirely new delivery concept, but rather to assess the practical benefit of adding folate-mediated targeting to an existing PEGylated liposomal Cel platform.

**Figure 1:**
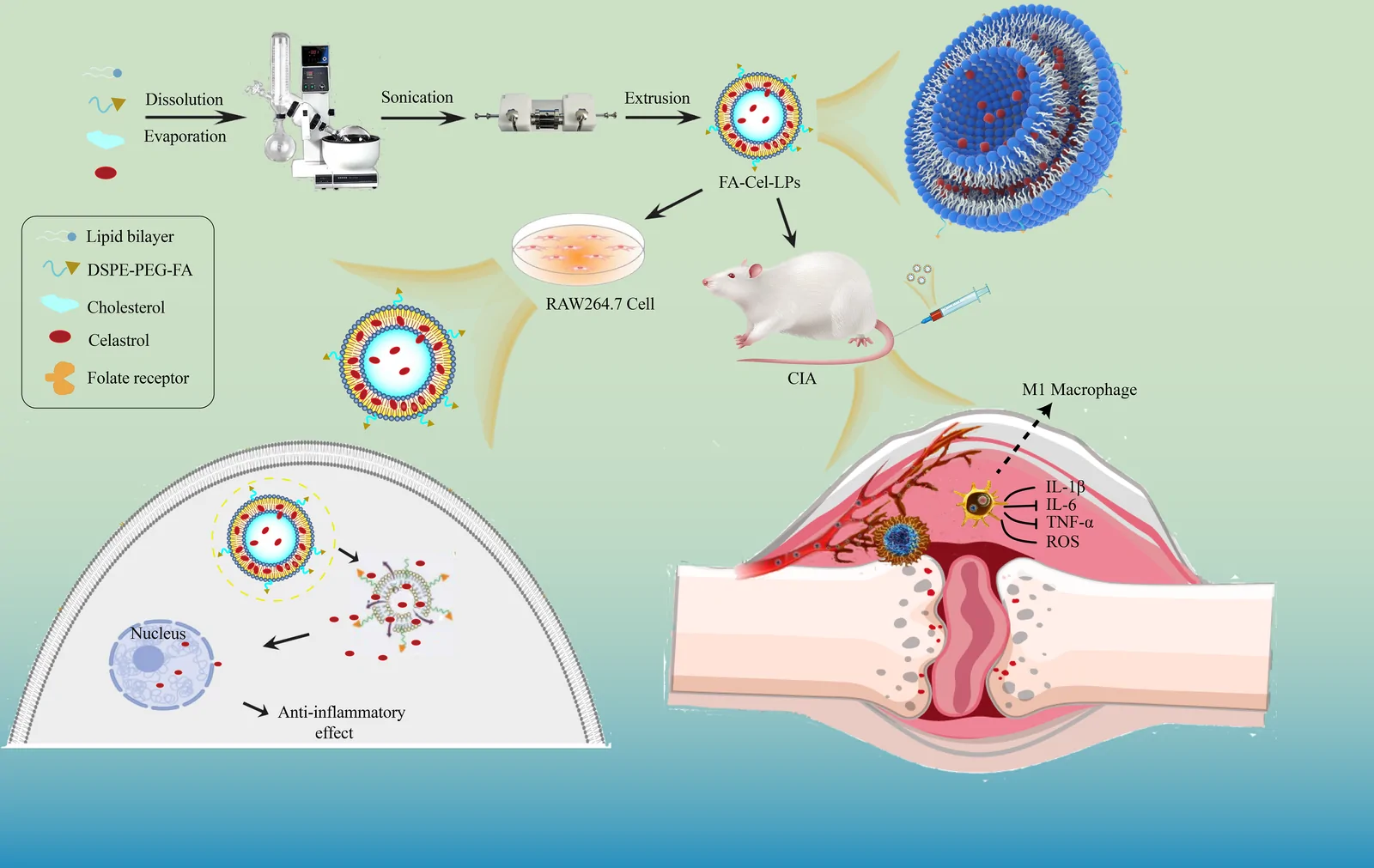
Schematic illustration of the preparation of FA-Cel-LPs, their proposed joint-targeting behavior in rheumatoid arthritis, and their potential involvement in ROS/NF-κB/COX-2-related inflammatory signaling.

In this context, folate-modified liposomes encapsulating Celastrol (FA-Cel-LPs) were developed to improve the solubility, formulation stability, and inflamed-joint accumulation of Cel. Their therapeutic effects were systematically evaluated in LPS-stimulated RAW264.7 macrophages and in CIA rats. In addition, changes associated with oxidative stress and inflammatory signaling were explored, with particular attention to the ROS/NF-κB/COX-2-related signaling (**Figure 1**).

## 2 Materials and methods

### 2.1 Materials

Celastrol (Cel, A0106–1 g, purity ≥ 99%) was purchased from Chengdu Must Bio-Technology Co., Ltd (Chengdu, China). Cholesterol (Chol), soy lecithin (SL), and folate acid (FA) were sourced from Shanghai Aladdin Biochemical Technology Co., Ltd (Shanghai, China). DSPE-PEG2000 was supplied by Shanghai Yare Biotechnology Co., Ltd (Shanghai, China), and DSPE-PEG2000-FA was obtained from Shanghai Yuanye Bio-Technology Co., Ltd (Shanghai, China). Rhodamine B (RhoB), DAPI (4′,6-diamidino-2-phenylindole), and DiR (1,1-dioctadecyl-3,3,3,3-tetramethylindotricarbocyanine iodide) were purchased from Beijing InnoChem Science & Technology Co., Ltd (Beijing, China). Primary antibodies against phosphorylated NF-κB p65 (p-p65, Cat#3303S), NF-κB p65 (p65, Cat#8242S), phosphorylated IκBα (p-IκBα, Cat#2859S), iNOS (Cat#13120S, Cat#20609S), COX-2 (Cat#12282S), β-actin (Cat#4967S), and normal rabbit IgG were acquired from Cell Signaling Technology (Beverly, MA, USA). Antibodies against TNF-α (Cat#60291-1-Ig) and IL-1β (Cat#16806-1-AP) were obtained from Proteintech (Rosemont, IL, USA). Dulbecco’s Modified Eagle’s Medium (DMEM), fetal bovine serum (FBS), penicillin–streptomycin (1%), trypsin-EDTA, and serum-free cryopreservation solution were provided by Gibco (Gaithersburg, MD, USA). The Cell Counting Kit-8 (CCK-8) and the Reactive Oxygen Species (ROS) Assay Kit were supplied by Shanghai Beyotime Biotech Inc. (Shanghai, China). The Cell Total RNA Extraction Kit was obtained from Chengdu Foregene Co., Ltd. (Chengdu, China). Complete Freund’s adjuvant (CFA) and lipopolysaccharides (LPS) were sourced from Sigma (St. Louis, MO). Bovine type II collagen was purchased from Chondrex, Inc (Redmond, WA, USA). The Hematoxylin and Eosin (H&E) Staining Kit was provided by Beijing Solarbio Science & Technology Co., Ltd (Beijing, China). All other reagents used were of analytical grade and commercially available.

### 2.2 Cells and animals

The murine macrophage cell line RAW264.7 was obtained from Wuhan Punosa Life Technology Co., Ltd. (Wuhan, China) and cultured in DMEM supplemented with 10% FBS and 1% penicillin-streptomycin at 37°C in a humidified atmosphere containing 5% CO_2_. Thirty healthy male Sprague-Dawley rats (220 ± 20 g) were provided by the Experimental Animal Center of Wenzhou Medical University (Wenzhou, China). The animals were housed under specific pathogen-free (SPF) conditions (25 ± 1°C, 60 ± 5% relative humidity, 12-h light/dark cycle) with ad libitum access to standard chow and water and were acclimatized for 7 days prior to the experiments.

### 2.3 Synthesis of FA-Cel-LPs

Cel-LPs and FA-Cel-LPs were formulated using the thin-film hydration and extrusion method. Briefly, 100 mg of soy lecithin (SL), 16.67 mg of cholesterol (Chol), and 8.33 mg of DSPE-PEG2000 were co-dissolved in a chloroform-methanol solvent mixture (15 mL, 2:1, v/v) containing 2.5 mg, 5 mg, or 10 mg of Cel. The mixture was vortexed thoroughly and evaporated under vacuum at 45°C using a rotary evaporator for 2 h to form a thin lipid film. The film was hydrated with 10 mL of phosphate-buffered saline (PBS, 0.01 M, pH 7.4) and subjected to ultrasonication for 30 min. The resulting suspension was sequentially extruded through polycarbonate membranes with pore sizes of 1000 nm, 450 nm, and 220 nm, producing Cel-LPs with three different drug-to-lipid mass ratios (Cel: SL ratios of 1:5, 1:10, and 1:20). Unencapsulated Cel and free lipids were removed by overnight dialysis against PBS using an 8–14 kDa molecular weight cut-off (MWCO) dialysis membrane. FA-modified Cel-LPs (FA-Cel-LPs) were prepared using the same procedure, substituting DSPE-PEG2000 entirely with DSPE-PEG2000-FA to achieve the same drug-to-lipid ratios (1:5, 1:10, and 1:20). Blank liposomes (Blank LPs and Blank FA-LPs) were prepared similarly without the addition of Cel. All liposome formulations were prepared in three independent batches for characterization.

### 2.4 Characterization of different liposomes

Encapsulation efficiency (EE) and drug-loading capacity (DLC) of Cel-LPs and FA-Cel-LPs prepared at different drug-to-lipid ratios were quantified using ultra-performance liquid chromatography-tandem mass spectrometry (UPLC–MS/MS) against an established celastrol calibration curve. Liposome suspensions were disrupted via ultrasonic homogenization, followed by centrifugation, and the celastrol content in the supernatant was determined to calculate EE and DLC. The optimal drug-to-lipid ratio was selected based on EE and DLC. Detailed UPLC-MS/MS conditions for celastrol quantification are provided in the **Supplementary Information** (S1. UPLC-MS/MS analysis of celastrol).

The hydrodynamic particle size (PS), polydispersity index (PDI), and zeta potential (ZP) of Blank LPs, Blank FA-LPs, Cel-LPs, and FA-Cel-LPs were measured by dynamic light scattering using a Zetasizer Nano ZS (Malvern Instruments, UK). Freshly prepared liposomal suspensions were diluted with an equal volume of deionized water prior to measurement to minimize multiple scattering effects. Particle size and PDI were determined by dynamic light scattering at 25°C, and zeta potential was measured by electrophoretic light scattering under the same conditions. Measurements were performed using three independently prepared batches of each formulation (n = 3). Stability studies were conducted by monitoring changes in PS, PDI and ZP of these four types of liposomes over two weeks at 4°C. The morphology and ultrastructure of FA-Cel-LPs were examined by transmission electron microscopy (TEM; FEI Tecnai G2 F20 S-TWIN, FEI, USA). Briefly, a drop of diluted liposome suspension was deposited onto a carbon-coated copper grid (300 mesh) and allowed to adsorb for 2 min. Excess liquid was removed with filter paper, and the sample was negatively stained with 2% (w/v) phosphotungstic acid for 1 min. After staining, excess stain was blotted away with filter paper, and the grid was allowed to air-dry at room temperature before TEM imaging.

*In vitro* drug release from FA-Cel-LPs was assessed using a dialysis method under two pH conditions (pH 7.4 and pH 6.5). Phosphate-buffered saline containing 0.5% (v/v) Tween 80 (PBST) was used as the release medium to maintain sink conditions and improve celastrol solubility. Freshly prepared FA-Cel-LPs (0.5 mL) were loaded into pre-activated dialysis bags (8–14 kDa MWCO) and immersed in 40 mL of PBST at 37 ± 1°C with continuous shaking at 100 rpm. Three parallel samples were prepared for each condition. At predetermined intervals (0.125, 0.25, 0.5, 1, 2, 4, 6, 8, 10, 24, 48, 72, 96, 120 and 144 h), 1 mL of the external medium was withdrawn and replaced with an equal volume of fresh PBST to maintain sink conditions. The Cel concentration in the withdrawn medium was analyzed by UPLC-MS/MS in the **Supplementary Information** (S1. UPLC-MS/MS analysis of celastrol), and the cumulative release was calculated accordingly. All formulation preparations and physicochemical characterizations were performed in independent triplicates (n = 3).

### 2.5 Cell viability assay

Cytotoxicity was assessed by the Cell Counting Kit-8 (CCK-8) assay. RAW264.7 cells were seeded in 96-well plates and cultured overnight in complete medium, then treated for 24 h with free celastrol (0.125, 0.25, 0.5, and 1 μg/mL) or equivalent celastrol concentrations of FA-Cel-LPs and Cel-LPs. For each treatment concentration, three replicate wells were used. Corresponding blank carriers (Blank FA-LPs and Blank LPs) were tested at matched lipid concentrations. After treatment, CCK-8 reagent (10 μL/well) was added and incubated at 37 °C for 1 h. Absorbance at 450 nm was measured using a microplate reader (Thermo Fisher Scientific), and cell viability was expressed relative to untreated controls. The experiment was repeated three times using independently seeded and treated cell cultures.

**Figure 2:**
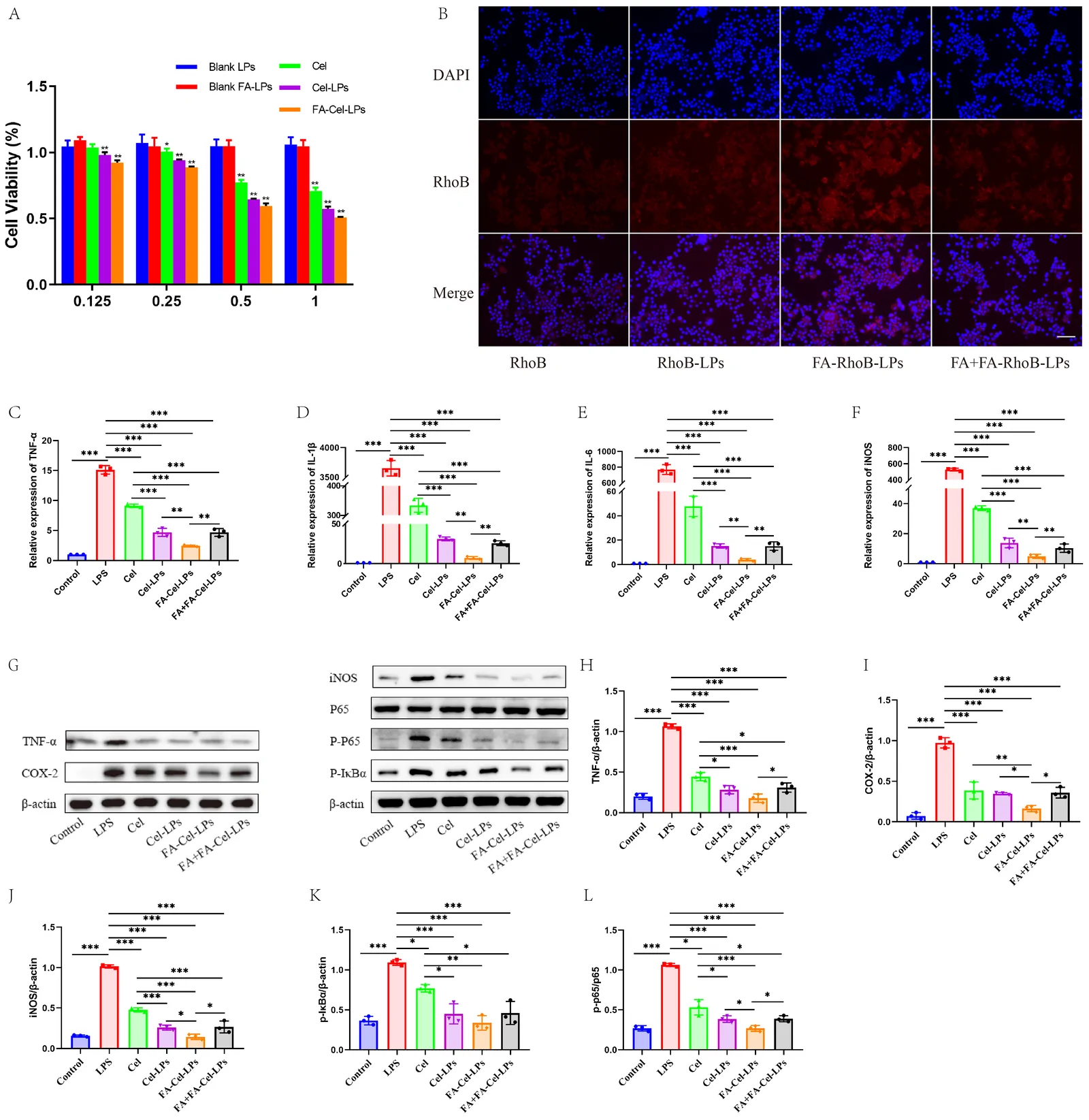
*In vitro* cytotoxicity, targeted cellular uptake, and anti-inflammatory effects of FA-Cel-LPs in RAW264.7 macrophages. **(A)** Cell viability after a 24-h incubation with increasing concentrations of free Cel, Cel-LPs, FA-Cel-LPs, Blank LPs, or Blank FA-LPs, assessed by the CCK-8 assay (n = 3 independent experiments). **(B)** Comparative uptake of free RhoB, RhoB-LPs, FA-RhoB-LPs, and FA-RhoB-LPs + free FA (competitive inhibition) after 4 h incubation in LPS-activated RAW264.7 macrophages (scale bar = 100μm). Blue: DAPI-stained nuclei; red: RhoB fluorescence. **(C-F)** Quantitative relative mRNA expression levels of TNF-α **(C)**, IL-1β **(D)**, IL-6 **(E)**, and iNOS **(F)** determined by qRT-PCR (n = 3 independent samples). **(G)** Western blot analysis of TNF-α, COX-2, iNOS, p-p65, p65 and p-IκBα protein expression in LPS-activated RAW264.7 cells following different treatments. **(H-L)** Densitometric analysis of the corresponding proteins. Relative expression of TNF-α normalized to β-actin **(H)**, COX-2 normalized to β-actin **(I)**, iNOS normalized to β-actin **(J)**, p-IκBα normalized to β-actin **(K)**, and p-p65 normalized to total p65 **(L)**. All results are shown as the mean ± SD. *p < 0.05, **p < 0.01, ***p < 0.001 indicate statistically significant differences between the designated groups.

Based on the preliminary cytotoxicity evaluation, 0.25 μg/mL Cel was selected for subsequent *in vitro* experiments (**Figure 2A**). Cells were assigned to the following groups: Control, LPS, Cel (0.25 μg/mL), Cel-LPs (equivalent to 0.25 μg/mL Cel), FA-Cel-LPs (equivalent to 0.25 μg/mL Cel), and FA+FA-Cel-LPs (equivalent to 0.25 μg/mL Cel). To assess folate receptor–mediated uptake, the FA+FA-Cel-LPs group was pre-treated with free folic acid (150 μg/mL) as a competitive inhibitor. Except for the Control group, all cells were stimulated with LPS (1 μg/mL) to induce a pro-inflammatory macrophage phenotype.

### 2.6 Cellular uptake

To visualize cellular uptake, fluorescent liposomes were prepared by replacing celastrol with rhodamine B (RhoB), yielding RhoB-LPs and FA-RhoB-LPs using the same formulation procedure. Unencapsulated RhoB was removed via ultracentrifugation, and the purified liposomal pellet was re-dispersed in serum-free DMEM. The particle size of the re-dispersed liposomes was re-measured and showed no obvious change compared with that measured before centrifugation, indicating acceptable colloidal stability after re-dispersion. The RhoB content in liposomes was quantified by fluorescence spectrophotometry. RAW264.7 cells were seeded in 12-well plates containing round glass coverslips and cultured overnight. After LPS activation, cells were incubated at 37°C with free RhoB (0.5 mg/mL), RhoB-LPs (equivalent to 0.5 mg/mL RhoB), FA-RhoB-LPs (equivalent to 0.5 mg/mL RhoB), or FA-RhoB-LPs plus free folic acid (competitive inhibition; equivalent to 0.5 mg/mL RhoB) for 4 h. Cells were fixed with 4% paraformaldehyde for 15 min and mounted using anti-fade medium containing DAPI. Fluorescence images were acquired using an inverted fluorescence microscope, and RhoB fluorescence was qualitatively compared across groups. The experiment was performed in three independent replicates, and representative images are shown.

### 2.7 Quantitative real-time PCR

After grouping and drug treatment, RAW264.7 cells were collected, and total RNA was extracted using the Cell Total RNA Extraction Kit. The RNA concentration and purity were assessed by measuring the A260/A280 ratio with a Nanodrop spectrophotometer. RNA was subsequently reverse-transcribed into cDNA using a reverse transcription reaction system. Quantitative real-time PCR (qRT-PCR) was performed using a three-step protocol (45 cycles): initial denaturation at 95 °C for 30 seconds, denaturation at 95°C for 10 seconds per cycle, and annealing/elongation at 56°C for 30 seconds. Each experimental group included three replicate wells. *GAPDH* was used as the internal control, and the relative mRNA expression of inflammatory cytokines was calculated using the 2^-ΔΔCt^ method. mRNA levels of inflammatory markers, including *TNF-α*, *IL-1β*, *IL-6*, and *iNOS*, were quantified. The primer sequences used are listed in **Table 1**. All experiments were performed in three independent biological replicates (n = 3 independent experiments).

**Table 1:** Primer sequences used in qRT-PCR.

Gene	Primer	Nucleotide sequences (5’-3’)
*TNF-α*	Forward	CACCACGCTCTTCTGTCTACTGAAC
	Reverse	TGGGCTACGGGCTTGTCAC
*iNOS*	Forward	TCTTGGAGCGAGTTGTGGATTGTTC
	Reverse	AGTGATGTCCAGGAAGTAGGTGAGG
*IL-1β*	Forward	AATCTCACAGCAGCATCTCGACAAG
	Reverse	TCCACGGGCAAGACATAGGTAGC
*IL-6*	Forward	ACTTCCAGCCAGTTGCCTTCTTG
	Reverse	TGGTCTGTTGTGGGTGGTATCCTC
*GAPDH*	Forward	GAAGCTGGTCATCAACGGGA
	Reverse	ACGACATACTCAGCACCAGC

### 2.8 Western blot analysis

RAW264.7 cells were grouped and treated as described previously. After 24 h of incubation, 150 μL of lysis buffer (RIPA supplemented with protease inhibitors, PMSF, and phosphatase inhibitors at a ratio of 100:1:1:10, v/v) was added to each well to extract cellular proteins. Protein concentrations were quantified using the BCA assay. The samples were then heated at 100°C for 5 min, cooled on ice, and stored at -80°C until analysis. Proteins were separated using 10% polyacrylamide gel electrophoresis (stacking gel: 70 V for 30 min; resolving gel: 90 V for 90 min). The separated proteins were transferred onto PVDF membranes at 280 mA for 90 min. Membranes were blocked with 5% skim milk for 2 h at room temperature on a shaker and washed three times for 5 min each with TBST (Tris-buffered saline with Tween 20). Primary antibodies specific to β-actin, TNF-α, COX-2, iNOS, p-p65, p65 and p-IκBα (all diluted 1:1000) were applied, and membranes were incubated overnight at 4°C. The next day, membranes were washed three times with TBST for 10 min each. Subsequently, HRP-conjugated anti-rabbit secondary antibody was added and incubated at 37°C for 2 h. After another three washes with TBST (10 min each), the membranes were incubated with ECL detection reagent (1:1 mixture of solutions A and B) for 2 min in the dark. Protein bands were visualized using the ChemiDoc Touch imaging system and quantitatively analyzed for target bands using Image J software. Each experiment was repeated three times using independent cell cultures (n = 3 independent experiments).

### 2.9 Immunofluorescence staining

RAW264.7 cells were seeded into 12-well plates containing round glass coverslips. After cell adhesion, interventions were performed according to the previously described grouping and treatment protocols, followed by 24 h of incubation. Cells were fixed with 4% paraformaldehyde for 15 min, then blocked with 3% BSA for 30 min. The primary antibody, IL-1β rabbit polyclonal antibody (diluted 1:200 using the primary antibody dilution buffer), was added and incubated overnight at 4°C in a humidified chamber. After incubation, the cells were equilibrated to room temperature for 30 min, followed by incubation with secondary antibody for 1 h at room temperature in the dark. Cy3-conjugated goat anti-rabbit IgG secondary antibody was used, diluted 1:200 in TBS. Then, cells were stained with DAPI. Fluorescence images were acquired using a fluorescence microscope under identical exposure conditions for all groups. The mean fluorescence intensity of IL-1β was quantified using ImageJ software. All experiments were repeated three times using independent cell cultures (n = 3 independent experiments).

### 2.10 Intracellular ROS assessment

Intracellular ROS levels were assessed using the DCFH-DA fluorescent probe method following various treatments. RAW264.7 cells were grouped and treated as previously described, with the inclusion of a positive control group treated with Rosup at a final concentration of 1 μL/mL. After treatment, cells were incubated in a 37°C culture incubator for 24 h. All groups were subsequently incubated with 10 μM DCFH-DA diluted in serum-free medium at 37°C for 30 min. Then, cells were stained with DAPI. The fluorescence intensity of DCF was examined using an inverted fluorescence microscope. Finally, quantitative analysis of fluorescence intensity was performed using ImageJ software. All experiments were independently repeated three times using separate cell cultures (n = 3 independent experiments).

### 2.11 Hemolysis assay

The blood biocompatibility of LPs, FA-LPs, Cel-LPs and FA-Cel-LPs was evaluated using an *in vitro* hemolysis assay. Whole blood (4 mL) was collected from anesthetized healthy rats via cardiac puncture, placed in heparinized anticoagulant tubes, and centrifuged at 2500 rpm for 10 min. The supernatant was discarded, and the erythrocytes were washed with 0.9% saline repeatedly until the supernatant remained colorless. The assay groups were prepared as follows (1): Negative control group (0% hemolysis): 40 μL of the diluted erythrocyte suspension was added to 960 μL of 0.9% physiological saline to obtain a 2% (v/v) erythrocyte suspension. (2) Positive control group (100% hemolysis): 40 μL of the diluted erythrocyte suspension was added to 960 μL of distilled water to obtain a 2% (v/v) erythrocyte suspension. (3) Experimental groups: 40 μL of the diluted erythrocyte suspension was added to 960 μL of LPs, FA-LPs, Cel-LPs, or FA-Cel-LPs sample solutions. For the drug-loaded liposomes, the equivalent Cel concentration was 0.1 mg/mL. All samples were incubated at 37°C for 2 h, followed by centrifugation at 3000 rpm for 10 min. Supernatants were collected, and absorbance at 540 nm was measured using a microplate reader. The hemolysis percentage was calculated using the standard formula after correcting for solvent interference (n = 3 independent replicates).

### 2.12 CIA model and treatment

Following a 7-day adaptive feeding period, thirty male Sprague-Dawley rats were randomly assigned to five groups (n=6 rats per group) based on the treatment regimen: Control group (treated with PBS), CIA group (CIA + PBS), Cel group (CIA + free Cel), Cel-LPs group (CIA + Cel-loaded liposomes), and FA-Cel-LPs group (CIA + folate-modified Cel-loaded liposomes). Except for the control group, all other groups underwent CIA modeling. To induce CIA, bovine type II collagen was emulsified with complete Freund’s adjuvant at a 1:1 volume ratio. For the primary immunization (Day 0), 200 µL of the emulsion was administered subcutaneously at the base of the tail. Seven days later (Day 7), a booster immunization of 100 µL was administered at the same site. One week after the booster (Day 14), arthritis severity was assessed using a standard arthritis scoring system: 0, no visible erythema or swelling; 1, mild redness and swelling in the ankle and wrist joints; 2, erythema with moderate swelling extending from the ankle to the mid-foot; 3, severe redness and swelling involving the claws, including fingertips; 4, maximum inflammation affecting multiple joints in the limbs. The Control group received equal volumes of physiological saline on Days 0 and 7. Starting on Day 14, the Cel, Cel-LPs, and FA-Cel-LPs groups were treated with 1 mg/kg Cel via sublingual vein injection every three days, for a total of 5 doses. The 1 mg/kg Cel dose used *in vivo* was selected based on published efficacy and tolerability data in CIA rodent models (11, 17, 19), which represents the maximum well-tolerated dose for free Cel in this model. The same equivalent Cel dose was used across free-Cel, Cel-LPs, and FA-Cel-LPs groups to enable direct comparison of delivery-system contributions.

**Figure 3:**
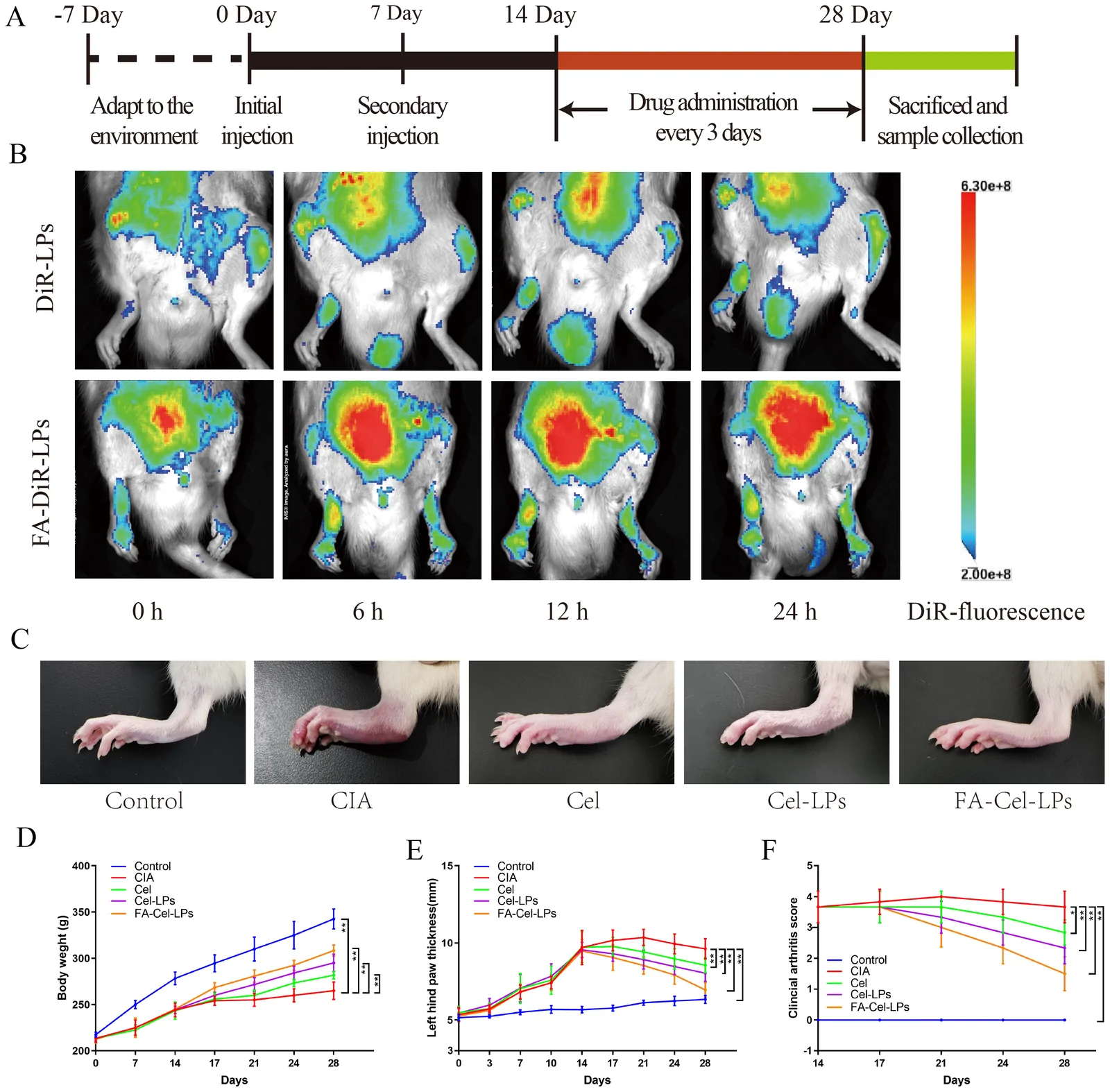
*In vivo* fluorescence imaging and therapeutic evaluation of FA-Cel-LPs in the CIA rat. **(A)** Schematic timeline of CIA induction and treatment protocol. Rats were immunized with bovine type II collagen on days 0 and 7; treatments were administered via the sublingual vein every three days from day14 to day26 (for a total of five doses). **(B)** Representative *in vivo* fluorescence images of CIA rats at 0, 6, 12, and 24 h after intravenous injection of DiR-LPs or FA-DiR-LPs. **(C)** Representative macroscopic images of left hind paws at the end of the experiment showing the extent of joint swelling. **(D)** Body weight changes of rats during the treatment period (n = 6 per group). **(E)** Left hind paw thickness measured every three days (n = 6 per group). **(F)** Arthritis scores recorded every three days (n = 6 per group). All results are shown as the mean ± SD. *p<0.05, **p<0.01, ***p<0.001 compared with the untreated CIA group.

The Control and CIA groups received equal volumes of PBS under the same schedule. The therapeutic efficacy was comprehensively evaluated through behavioral assessment, imaging studies, organ index analysis, histopathological examination, serum biochemical analysis and *in vivo* fluorescence imaging (**Figure 3A**).

### 2.13 Behavioral evaluation and experimental timeline

After group allocation, body weight, survival status, overall activity levels, and food intake of the rats were monitored every three days. The thickness of the left hind paw was measured using a digital vernier caliper every three days to quantify swelling. Arthritis severity was simultaneously evaluated at the same interval using the arthritis score scoring system. Prior to euthanasia, magnetic resonance imaging (MRI) of the knee joints was conducted, and macroscopic photographs of the left hind paws were taken. For live-animal procedures requiring immobilization, inhalational isoflurane anesthesia was administered. Forty-eight hours after the final administration, the rats were euthanized. Briefly, rats were deeply anesthetized with an intraperitoneal injection of sodium pentobarbital (100 mg/kg body weight). Once surgical anesthesia was confirmed by the absence of the pedal reflex, blood samples were collected via cardiac puncture, followed by exsanguination to ensure humane euthanasia. The left knee joints were promptly harvested for micro-computed tomography (micro-CT) analysis, and major organs were preserved for histopathological examination.

### 2.14 *In vivo* fluorescence imaging

To evaluate the targeting efficiency and biodistribution of FA-Cel-LPs, DiR was utilized as a near-infrared fluorescent probe for a non-invasive optical imaging system. DiR-LPs and FA-DiR-LPs were prepared following the previously described methodologies and intravenously administered to CIA rats (n=3 per group) via the sublingual vein at a DiR dose equivalent to 400 μg/kg. Rats were anesthetized with isoflurane inhalation, and fluorescence images were captured at 0, 6, 12, and 24 h post-injection using the IVIS Lumina III *In Vivo* Imaging System (PerkinElmer Inc., Waltham, MA, USA).

### 2.15 Micro-CT analysis

The left knee joints of three randomly selected rats per group (n=3) were subjected to immediate micro-CT imaging (Skyscan 1176, Bruker, Belgium) to assess changes in trabecular and cortical bone architecture. Following euthanasia, the left knee joints were carefully excised; surrounding soft tissues were meticulously removed while ensuring the integrity of the joint capsule. The samples were fixed in 4% paraformaldehyde. Each knee joint was positioned vertically in the sample chamber, with the scanning parameters set to a tube voltage of 60 kV and a tube current of 120 µA. During the scan, the detector and X-ray source rotated 360° around the central axis of the sample chamber, acquiring 4000 projections over a 1200-second scanning period. The raw data were transmitted to a computer and reconstructed using the Feldkamp-Davis-Kress (FDK) algorithm with Avatar software, producing the final high-resolution three-dimensional images. Bone volume fraction (BV/TV, defined as the ratio of bone volume to total tissue volume, %), bone surface to tissue volume ratio (BS/TV, defined as the bone surface area normalized to total tissue volume, mm^-1^), and bone mineral density (BMD, defined as the mineral content per unit bone volume, g/cm^3^) were calculated.

### 2.16 MRI analysis

The left knee joints of three randomly selected rats per group (n=3) were examined using MRI to evaluate joint effusion, synovial membrane hyperplasia, and cartilage integrity. Rats were anesthetized with an intraperitoneal injection of sodium pentobarbital (50 mg/kg body weight). The depth of anesthesia was monitored by the absence of the pedal reflex throughout the imaging procedure, with additional maintenance doses (10 mg/kg) administered if necessary. Once anesthetized, rats were positioned in a specialized small-animal coil (Shanghai Chenguang, China) to ensure maximum knee extension. Conventional sagittal T2-weighted imaging (T2WI) was performed using a GE Discovery MR750 3.0T scanner with the following parameters: TR/TE = 1800 ms/80 ms, slice thickness = 3 mm, interslice gap = 0.5 mm, number of slices = 6, matrix size = 192 × 192, field of view (FOV) = 5.0 cm, and scan duration = 110 s. One representative image from each group was selected for display, and key slices from all sequences were systematically analyzed.

### 2.17 Histology and immunohistochemical study

After euthanasia, intact knee joints were collected from all rat groups. Surface muscles and skin were carefully removed, and the joints were fixed in 10% neutral buffered formalin for 3 days, followed by decalcification in 10% EDTA solution for approximately 2 months. The decalcified joints were progressively dehydrated, embedded in paraffin, and sectioned at a thickness of 4-5 μm. Histological evaluation was performed using H&E staining to assess synovial cell infiltration, cartilage degradation, and bone erosion. Immunohistochemical (IHC) staining was conducted for COX-2 and iNOS. Paraffin sections were dewaxed with xylene, rehydrated through a graded ethanol series, and subjected to heat-induced antigen retrieval using sodium citrate buffer. Sections were washed with PBS and blocked with 5% goat serum for 20 min. Primary antibodies against COX-2 (diluted 1:200) and iNOS (diluted 1:200) were applied, followed by overnight incubation at 4°C. After three PBS washes, sections were incubated with an HRP-conjugated anti-rabbit secondary antibody for 1 h at room temperature. Following additional rinses, a DAB substrate solution was applied for 3 min, and sections were counterstained with hematoxylin. Staining results were visualized under an optical microscope to evaluate protein expression levels in relation to pathological changes.

### 2.18 Organ index and histopathological staining

Following euthanasia, the heart, liver, spleen, lungs, kidneys, and thymus were carefully excised from the rats. The organs were rinsed with physiological saline, blotted dry with filter paper, and accurately weighed. The relative organ weight (organ index) was calculated as the ratio of organ weight to body weight (mg/g). Subsequently, all harvested organs were fixed in 10% formalin, sectioned, and stained with H&E. The stained sections were examined microscopically to assess potential systemic toxicity and histopathological changes.

### 2.19 Serum biochemical analysis

At the end of the treatment, whole blood was collected from the hearts of rats under anesthesia. Serum was separated by centrifugation (5000 × g, 10 min). The levels of total protein (TP), albumin (ALB), total bilirubin (TBIL), aspartate aminotransferase (AST), alanine aminotransferase (ALT), blood urea nitrogen (BUN), and creatinine (CREA) in the serum were automatically measured using a fully automated animal-specific biochemical analyzer (Celercare V5S) according to the manufacturer’s protocol. AST and ALT levels were assessed to evaluate the impact of the drugs on liver function, while BUN and CREA levels were measured to assess the impact on kidney function.

### 2.20 Randomization, blinding, and replicates

Rats were randomly assigned to experimental groups using a computer-generated random number sequence. Arthritis scoring, micro-CT analysis, MRI evaluation, and histological assessment were performed by two independent investigators blinded to group allocation. All *in vitro* experiments were performed with three biological replicates and three technical replicates per condition unless otherwise specified.

### 2.21 Statistical analysis

All quantitative data are presented as the mean ± standard deviation (SD). Statistical analyses and graphical representations were performed using GraphPad Prism (version 8.0, GraphPad Software, San Diego, CA, USA) and SPSS (version 22.0, SPSS Inc., Chicago, IL, USA). The normal distribution of data was first assessed using the Shapiro-Wilk test. For comparisons between two groups, a two-tailed Student’s t-test was used. For multiple group comparisons, one-way analysis of variance (ANOVA) was performed, followed by the Student-Newman-Keuls (SNK) *post-hoc* test for group comparisons. Time-course data (e.g., body weight, paw thickness, and arthritis scores) were analyzed using two-way repeated-measures ANOVA, followed by appropriate *post hoc* multiple-comparison tests. Values of *p* < 0.05 were considered statistically significant.

## 3 Results

### 3.1 Preparation and physicochemical characterization of liposomal formulations

**Figure 4:**
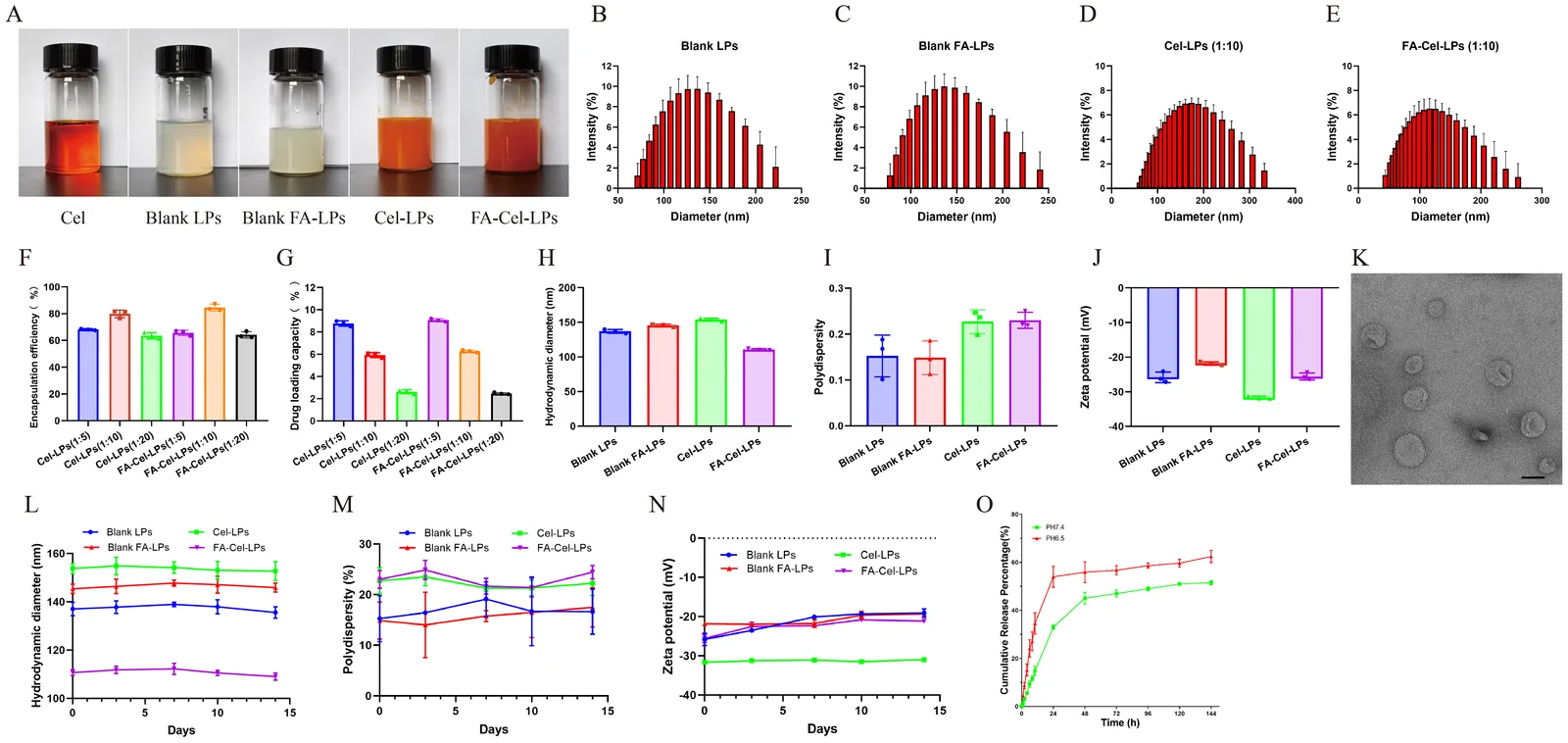
Physicochemical characterization of different liposomal formulations. **(A)** Digital photographs of free Cel dissolved in DMSO, Blank LPs, Blank FA-LPs, Cel-LPs, and FA-Cel-LPs. **(B-E)** Particle size distribution of Blank LPs **(B)**, Blank FA-LPs **(C)**, Cel-LPs (1:10) **(D)**, and FA-Cel-LPs (1:10) **(E)**. **(F)** Encapsulation efficiency **(EE)** and **(G)** drug loading capacity (DLC) of Cel-LPs and FA-Cel-LPs prepared at drug-to-lipid ratios of 1:5, 1:10, and 1:20 (n = 3 independent formulation preparations). **(H-J)** Average particle size **(H)**, polydispersity index (PDI, **(I)**), and zeta potential **(J)** of Blank LPs, Cel-LPs (1:10), Blank FA-LPs, and FA-Cel-LPs (1:10) (n = 3). **(K)** Transmission electron microscopy image of FA-Cel-LPs showing spherical morphology and uniform size (scale bar = 100 nm). **(L-N)** Storage stability of the four liposomal formulations at 4 °C over 14 days, monitored by particle size **(L)**, PDI **(M)**, and zeta potential **(N)** (n = 3 independent formulation preparations). **(O)** *In vitro* drug release profiles of FA-Cel-LPs in PBS (containing 0.5% Tween 80) at pH 7.4 and pH 6.5 over 144 h. All results are shown as the mean ± SD (n = 3).

The physicochemical properties of the engineered liposomal formulations are summarized in **Figure 4**. The macroscopic appearance of freshly prepared free Cel dissolved in DMSO, Blank LPs, Blank FA-LPs, Cel-LPs, and FA-Cel-LPs is shown in **Figure 4A**. The free Cel solution appeared as a clear, transparent reddish-brown liquid. In contrast, Blank LPs and Blank FA-LPs exhibited a milky white appearance. Both Cel-LPs and FA-Cel-LPs presented as homogeneous reddish-brown suspensions with no visible precipitation. Although slightly more turbid than the free Cel solution, the absence of precipitates indicated that liposomal encapsulation helped overcome the poor aqueous solubility of Cel. The PS distribution of each nanoparticle is shown in **Figures 4B–E**. To optimize the formulation, FA-Cel-LPs and Cel-LPs were prepared at drug-to-lipid mass ratios of 1:5, 1:10, and 1:20, and evaluated based on their encapsulation efficiency (EE) and drug loading capacity (DLC) (**Figures 4F, G**; **Table 2**). Both FA-Cel-LPs and Cel-LPs demonstrated higher loading parameters at a ratio of 1:10, with EE values of 84.67 ± 2.55% and 79.94 ± 2.88%, and DLC values of 6.27 ± 0.11% and 5.92 ± 0.22%, respectively. Therefore, the 1:10 ratio was selected for subsequent experiments based on its favorable EE and DLC.

**Table 2:** Encapsulation efficiency and drug loading capacity of FA-Cel-LPs and Cel-LPs at various drug-to-lipid ratios.

Sample	Ratio of drug to lipid	EE (%)	DL (%)
Cel-LPs	1:5	68.23 ± 0.60	8.76 ± 0.24
	1:10	79.94 ± 2.88	5.92 ± 0.22
	1:20	63.54 ± 2.22	2.62 ± 0.18
FA-Cel-LPs	1:5	65.74 ± 2.06	9.07 ± 0.14
	1:10	84.67 ± 2.55	6.27 ± 0.11
	1:20	64.18 ± 2.40	2.47 ± 0.08

All the results are shown as the mean ± SD (n = 3). EE, encapsulation efficiency; DLC, drug loading capacity.

The hydrodynamic PS, PDI, and ZP of Blank LPs, Blank FA-LPs, Cel-LPs (1:10), and FA-Cel-LPs (1:10) are detailed in **Figures 4H-J** and **Table 3**. The average PS were 137.05 ± 2.77 nm for Blank LPs, 145.37 ± 1.93 nm for Blank FA-LPs, 153.75 ± 2.13 nm for Cel-LPs, and 110.65 ± 1.24 nm for FA-Cel-LPs. The corresponding PDI values were 0.153 ± 0.045, 0.149 ± 0.037, 0.227 ± 0.026 and 0.230 ± 0.018, respectively, indicating relatively uniform size distributions. ZP values were measured as -25.82 ± 1.54 mV for Blank LPs, -21.81 ± 0.45 mV for Blank FA-LPs, -31.68 ± 0.40 mV for Cel-LPs, and -25.57 ± 1.06 mV for FA-Cel-LPs. TEM images revealed that FA-Cel-LPs showed spherical or oval vesicles with distinct lipid bilayer boundaries and a relatively uniform morphology (**Figure 4K**).

**Table 3:** Physical and pharmaceutical characterization of different nanoparticle formulations.

Formulation	PS (nm)	PDI	ZP (mV)
Blank LPs	137.05 ± 2.77	0.153 ± 0.045	-25.82 ± 1.54
Cel-LPs	153.75 ± 2.13	0.227 ± 0.026	-31.68 ± 0.40
Blank FA-LPs	145.37 ± 1.93	0.149 ± 0.037	-21.81 ± 0.45
FA-Cel-LPs	110.65 ± 1.24	0.230 ± 0.018	-25.57 ± 1.06

All the results are shown as the mean ± SD (n = 3). Particle size; PDI, polydispersity index; ZP, zeta potential.

The colloidal stability of the four liposomal formulations at 4°C over two weeks was assessed by monitoring PS, PDI, and ZP, as shown in **Figures 4L–N**. No significant changes were observed in these parameters, and no significant differences were found between groups, suggesting acceptable storage stability with limited aggregation during the observation period. Furthermore, the *in vitro* drug release profile of FA-Cel-LPs exhibited a pH-responsive pattern, as illustrated in **Figure 4O**. During the initial stage, rapid drug release occurred under both pH conditions, with 14.73 ± 1.91% of the drug released at physiological pH 7.4 and 34.52 ± 4.41% at slightly acidic pH 6.5 within the first 10 h. Around 120 h, the release plateaued, reaching cumulative release percentages of 50.96 ± 0.20% at pH 7.4 and 59.64 ± 1.67% at pH 6.5. The faster release rate and higher cumulative release at pH 6.5 suggest that an acidic environment may facilitate Cel release from FA-Cel-LPs *in vitro*.

### 3.2 *In vitro* cytotoxicity and biocompatibility

The cytotoxicity of Cel, Cel-LPs, FA-Cel-LPs, LPs, and FA-LPs on RAW264.7 macrophages was assessed using the CCK-8 assay. As shown in **Figure 2A**, LPs and FA-LPs exhibited negligible effects on cell viability, suggesting good *in vitro* biocompatibility of the lipid carriers under the tested conditions. After a 24 h exposure, cells treated with free Cel, Cel-LPs, and FA-Cel-LPs at 0.125–0.25 μg/mL maintained viability above 90%, suggesting minimal cytotoxicity within this concentration range. In contrast, cell viability decreased markedly at a Cel concentration of 0.5 μg/mL and declined further at higher doses, demonstrating dose-dependent cytotoxicity. Based on these results, a sub-toxic dose of 0.25 μg/mL Cel (or an equivalent Cel dose in liposomal formulations) was selected for subsequent *in vitro* assays.

### 3.3 Cellular uptake of folate-modified liposomes *in vitro*

To evaluate cellular internalization and folate-mediated targeting, the uptake of free rhodamine B (RhoB), RhoB-LPs, FA-RhoB-LPs, and FA-RhoB-LPs following folic acid competition (FA+FA-RhoB-LPs) was examined by fluorescence microscopy in LPS-activated RAW264.7 cells (**Figure 2B**). Compared with free RhoB, both RhoB-LPs and FA-RhoB-LPs exhibited stronger intracellular red fluorescence, indicating enhanced cellular internalization after liposomal encapsulation. The fluorescence signal was mainly distributed in the cytoplasm and perinuclear region. Notably, FA-RhoB-LPs showed stronger fluorescence intensity than non-targeted RhoB-LPs at the same RhoB concentration, suggesting that folate modification promoted the uptake of liposomes by activated macrophages. In contrast, pre-incubation with free folic acid reduced the intracellular fluorescence of FA-RhoB-LPs, supporting the involvement of a folate receptor-mediated uptake process.

### 3.4 FA-Cel-LPs reduce pro-inflammatory cytokine expression in LPS-activated macrophages

The impact of different drug formulations on the expression of inflammatory cytokine mRNA in LPS-induced RAW264.7 cells was assessed using qRT-PCR (**Figures 2C–F**). Compared to the control group, the mRNA levels of key pro-inflammatory factors, including *TNF-α*, *IL-1β*, *IL-6*, and *iNOS*, were significantly elevated in the LPS group. Treatment with all Cel-containing formulations significantly decreased the expression of these pro-inflammatory cytokines. Notably, the FA-Cel-LPs group exhibited the most pronounced reduction. Importantly, the expression levels in the receptor-blocked FA+FA-Cel-LPs group reverted to levels comparable to those in the non-targeted Cel-LPs group, while the free Cel group showed the least reduction. These findings suggest that FA-Cel-LPs exhibited the strongest inhibitory effect on pro-inflammatory cytokine expression among all treatment groups, and that folate receptor-mediated uptake may have contributed to this enhanced anti-inflammatory activity at the cellular level.

### 3.5 FA-Cel-LPs reduce NF-κB/COX-2/iNOS-related inflammatory signaling

The impact of various treatments on NF-κB/COX-2/iNOS pathway proteins in LPS-activated RAW264.7 cells was evaluated using Western blot analysis (**Figure 2G**). Compared to the control group, the LPS group exhibited significantly elevated levels of pro-inflammatory mediators (TNF-α, COX-2, iNOS) and key phosphorylation markers (p-IκBα and p-p65), while total p65 levels remained unchanged. Treatment with Cel, Cel-LPs, FA-Cel-LPs, and FA+FA-Cel-LPs reduced the expression of TNF-α, COX-2, p-p65, and p-IκBα induced by LPS stimulation. Consistent with the qRT-PCR results, the FA-Cel-LPs group achieved the most pronounced suppression of these pathway proteins. Notably, pre-treatment with free FA competitively inhibited this effect in the FA+FA-Cel-LPs group, with expression levels approaching those observed in the Cel-LPs group. These findings indicate that FA-Cel-LPs were associated with reduced NF-κB signaling activation and lower expression of downstream inflammatory mediators. Densitometric analysis (**Figures 2H–L**) further confirmed that LPS markedly activated this inflammatory pathway, as reflected by increases in the p-p65/p65 ratio and in COX-2 and iNOS expression relative to the control group. Treatment with FA-Cel-LPs significantly attenuated these LPS-induced changes (all *P* < 0.001 vs. LPS) and showed greater suppression than non-targeted Cel-LPs (*P* < 0.05).

### 3.6 Immunofluorescence analysis of intracellular IL-1β expression

**Figure 5:**
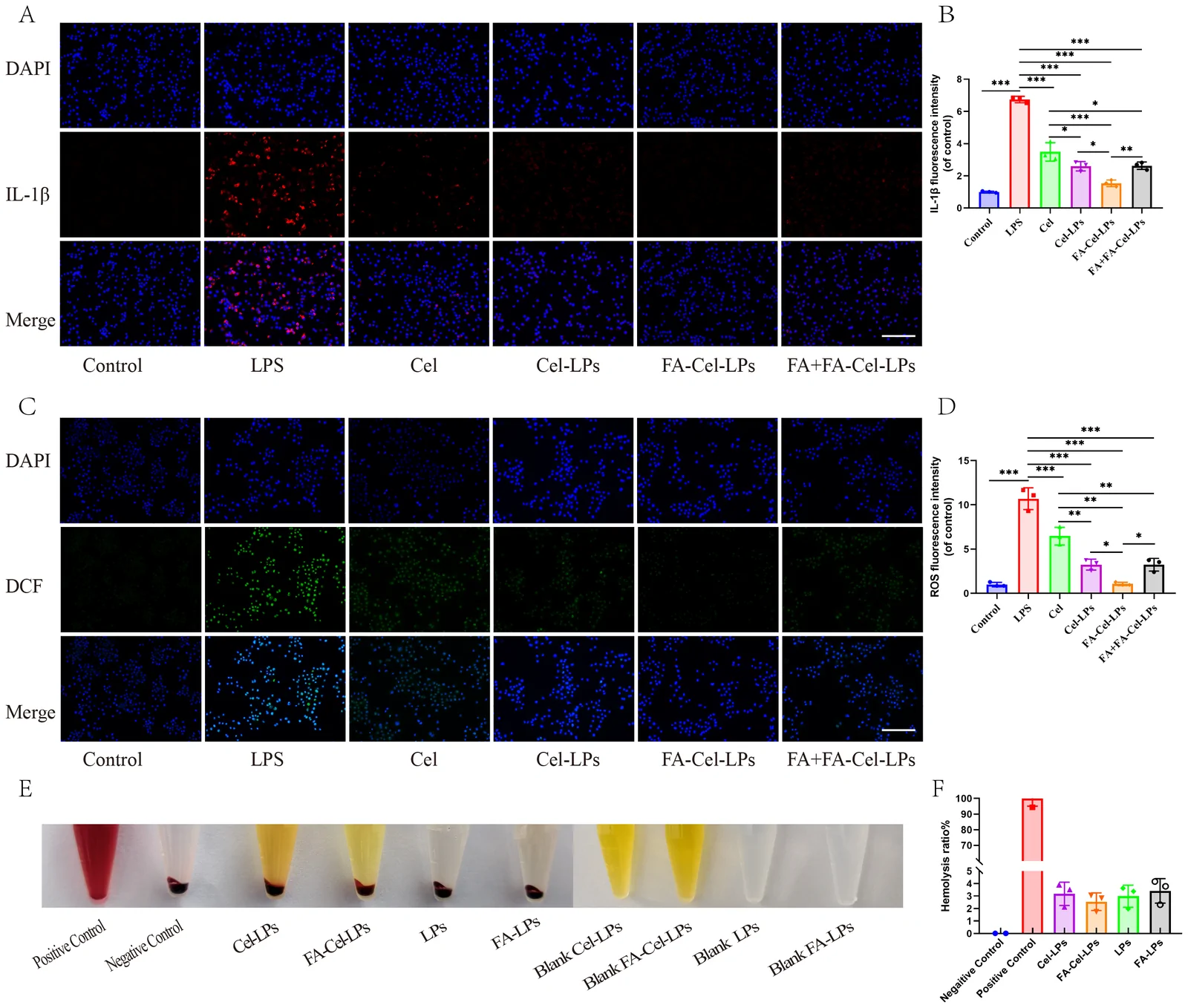
Effects of FA-Cel-LPs on pro-inflammatory IL-1β expression and intracellular oxidative stress in LPS-stimulated RAW264.7 cells. **(A)** Representative immunofluorescence images of IL-1β (red) in RAW264.7 cells. Nuclei were counterstained with DAPI (blue), Scale bars, 100μm. **(B)** Quantification of IL-1β fluorescence intensity. **(C)** Representative fluorescence images of intracellular ROS in RAW264.7 cells, detected by the DCFH-DA probe (green). Nuclei were counterstained with DAPI (blue). Scale bars, 100μm. **(D)** Quantification of ROS fluorescence intensity. Data in **(B, D)** are presented as the mean±SD (n=3 independent samples). **(E, F)** Hemolysis assay of LPs, FA-LPs, Cel-LPs, and FA-Cel-LPs. **(E)** Representative photograph of erythrocyte suspensions after incubation with different formulations and subsequent centrifugation. **(F)** Quantitative analysis of hemolysis rates for LPs, FA-LPs, Cel-LPs, and FA-Cel-LPs (n = 3 independent replicates). *p < 0.05, **p < 0.01, ***p < 0.001 indicate statistically significant differences between the designated groups.

The intracellular distribution and expression of the pro-inflammatory cytokine IL-1β (red fluorescence) were visualized using fluorescence microscopy (**Figure 5A**). Compared to the negligible signal in the control group, the fluorescence intensity of IL-1β significantly increased in the LPS-activated group. Following various drug treatments, fluorescence intensity decreased across all groups. Among these, the FA-Cel-LPs group exhibited the most dramatic reduction in IL-1β signal. The Cel-LPs group demonstrated a moderate decrease, while the fluorescence intensity in the receptor-blocked FA+FA-Cel-LPs group was comparable to that of the non-targeted Cel-LPs group. The free Cel group exhibited the least reduction in fluorescence intensity, suggesting that liposomal delivery, particularly with folate modification, was associated with greater inhibition of IL-1β expression at the cellular level. Quantitative analysis further confirmed the imaging results: IL-1β fluorescence intensity increased from 1.00 ± 0.04 in the control group to 6.74 ± 0.16 in the LPS group, and was reduced to 3.49 ± 0.47, 2.60 ± 0.24, and 1.54 ± 0.17 by free Cel, Cel-LPs, and FA-Cel-LPs, respectively. The receptor-blocked FA+FA-Cel-LPs group showed a higher fluorescence intensity (2.63 ± 0.19), comparable to that of Cel-LPs (**Figure 5B**).

### 3.7 FA-Cel-LPs attenuate intracellular oxidative stress (ROS generation)

Intracellular ROS levels were quantified using the ROS-sensitive probe DCFH-DA, which is oxidized to highly fluorescent DCF in the presence of reactive oxygen species. As shown in **Figure 5C**, the LPS group demonstrated a significant increase in green fluorescence intensity compared to the control group, indicating elevated ROS production under inflammatory stress. After drug treatment, fluorescence intensity decreased across all groups. Consistent with previous findings, the FA-Cel-LPs group exhibited the most substantial reduction in DCF fluorescence, indicating superior efficacy in scavenging intracellular ROS. The Cel-LPs group showed a moderate decrease, while the FA+FA-Cel-LPs group displayed a similar reduction to that observed in the Cel-LPs group. The free Cel group achieved the least reduction. These findings suggest that FA-Cel-LPs were associated with more effective attenuation of intracellular oxidative stress in this cell model. Quantitative analysis of DCF fluorescence intensity showed that LPS stimulation increased intracellular ROS to 10.7 ± 1.0-fold of the control level. This elevation was reduced to 6.5 ± 0.8-fold by free Cel, 3.2 ± 0.5-fold by Cel-LPs, and 1.1 ± 0.2-fold by FA-Cel-LPs. Notably, FA preincubation weakened this effect, with ROS remaining at 3.2 ± 0.6-fold of control in the FA+FA-Cel-LPs group (**Figure 5D**).

### 3.8 *In vitro* hemocompatibility of liposomal formulations

The hemolysis results of blank and Cel-loaded liposomal formulations are presented in **Figures 5E, F**. After incubation of the 2% erythrocyte suspension with LPs, FA-LPs, Cel-LPs, or FA-Cel-LPs, no obvious change in the color of the supernatant was observed compared with the negative control. After centrifugation, the erythrocyte pellets in all formulation-treated groups showed similar color and volume to those in the negative control group, indicating less hemolysis (**Figure 5E**). Quantitative analysis based on the absorbance of the supernatant at 540 nm showed that the hemolysis rates were 2.99 ± 0.57% for LPs, 3.42 ± 0.80% for FA-LPs, 3.18 ± 0.76% for Cel-LPs, and 2.55 ± 0.57% for FA-Cel-LPs. All values were below the generally accepted safety threshold of 5%, indicating good *in vitro* hemocompatibility of both blank and Cel-loaded liposomal formulations and supporting their preliminary suitability for intravenous administration.

### 3.9 *In vivo* fluorescence imaging of joint-associated accumulation

To evaluate whether FA modification influenced *in vivo* localization in inflamed joints, CIA rats were intravenously injected with DiR-labeled FA-LPs (FA-DiR-LPs) or non-targeted LPs (DiR-LPs). As shown in the representative images in **Figure 3B**, FA-DiR-LPs exhibited visually stronger and more persistent fluorescence signals in the inflamed hind paw region than non-targeted DiR-LPs during the observation period. These findings provide qualitative support for enhanced localization of FA-modified liposomes in inflamed joints. This distribution pattern suggests increased accumulation of FA-modified liposomes in inflamed joints. Furthermore, the fluorescence intensity of both formulations remained stable beyond 24 h, indicating sustained *in vivo* retention during the imaging period.

### 3.10 Therapeutic efficacy and systemic evaluation in CIA rats

The overall therapeutic efficacy was evaluated by monitoring clinical symptoms and physical signs. Prior to the experiment, all rats were healthy, displaying normal feeding and activity levels. By Day 14, the CIA group showed significant hind paw swelling, along with symptoms of depression, reduced food intake, and limited mobility compared to the control group. After two weeks of treatment, symptoms improved across all therapeutic groups, and no abnormal mortality occurred. Representative photographs of the left hind limb (**Figure 3C**) revealed severe joint swelling and deformity in the untreated CIA group, which were alleviated to varying extents by treatment with Cel, Cel-LPs, and FA-Cel-LPs.

Changes in body weight over four weeks are shown in **Figure 3D**. During the first week post-immunization, body weights were similar across all groups. From the second week onward, the CIA group exhibited a significant reduction in weight gain, with negligible increases by Day 14. In contrast, the FA-Cel-LPs group showed a greater recovery in body weight than the other treatment groups, while the Cel-LPs and Cel groups showed smaller increases.

Measurements of left hind paw thickness revealed that swelling peaked at Day 14 in the CIA group and remained elevated throughout the study (**Figure 3E**). All treatment groups demonstrated a reduction in swelling, with the FA-Cel-LPs group showing the most pronounced decrease, highlighting its superior efficacy in mitigating local inflammation. Similarly, the arthritis score remained high in the CIA group (**Figure 3F**). After treatment, arthritis scores gradually decreased in all treatment groups, with the FA-Cel-LPs group showing the greatest reduction, followed by the Cel-LPs group. The Cel group showed the least improvement. Collectively, these behavioral and physical assessments suggest that FA-Cel-LPs were associated with greater therapeutic benefit than free Cel and non-targeted liposomes in CIA rats.

### 3.11 Micro-CT evaluation of bone erosion and joint architecture

**Figure 6:**
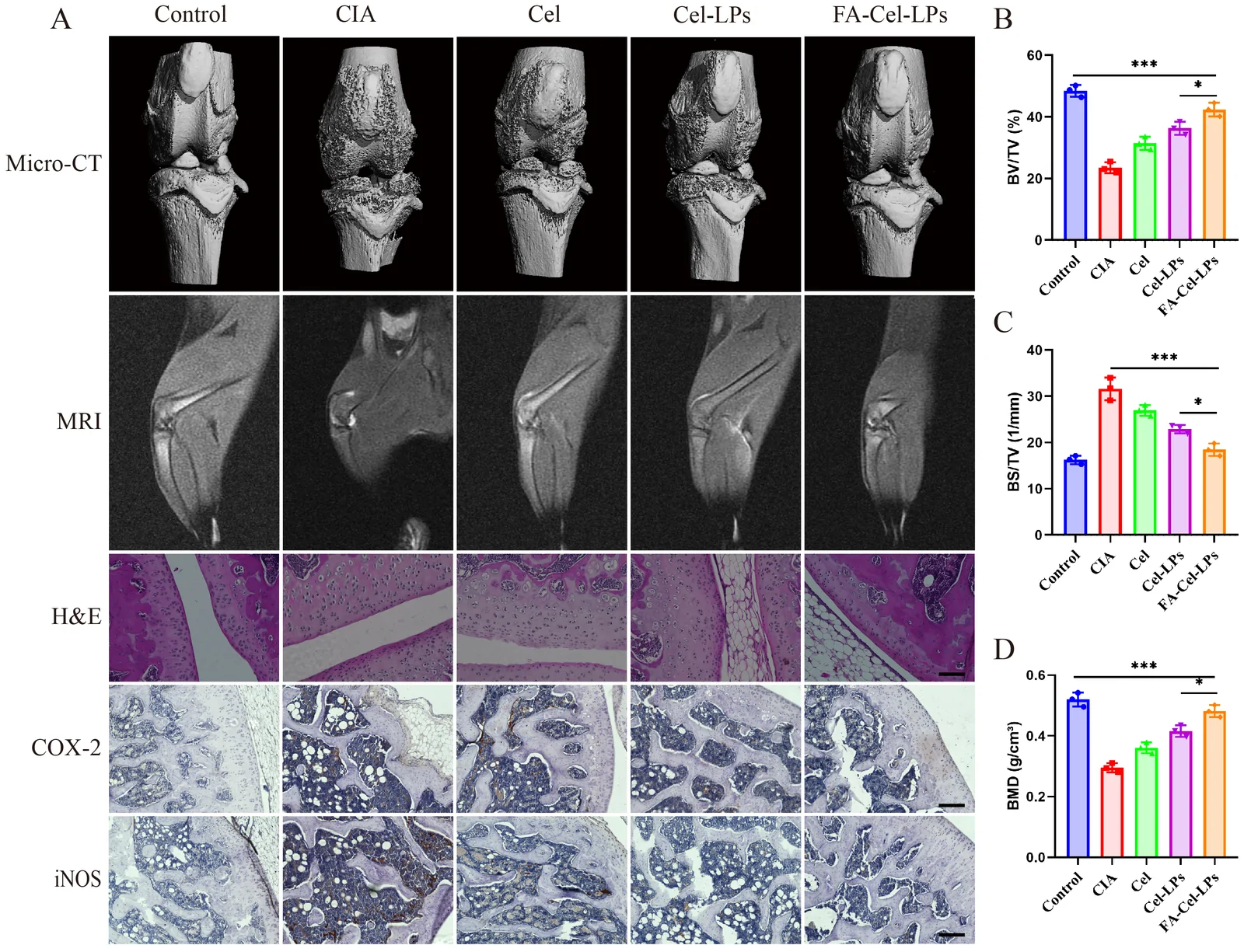
High-resolution imaging and histopathological evaluation of joint protection. **(A)** Representative images of left knee joints from each group. From top to bottom, panel A shows micro-CT images evaluating trabecular and cortical bone architecture, sagittal T2-weighted MRI images showing synovial hyperplasia and joint effusion, H&E-stained knee joint sections showing synovial inflammation (pannus formation), cartilage degradation, and bone erosion (scale bar = 100 μm), and immunohistochemical staining of COX-2 and iNOS in knee joint sections (scale bar = 100 μm). For imaging analyses, n = 3 rats per group. **(B-D)** Quantitative micro-CT analysis of distal femoral trabecular bone within the knee joint region. **(B)** BV/TV (%), **(C)** BS/TV (mm^-1^), and **(D)** BMD (g/cm^3^) of the distal femoral trabecular bone. Data are presented as the mean ± SD, n = 3. *p<0.05, ***p<0.001 indicate statistically significant differences between the designated groups.

Micro-CT images of the left knee joints are shown in **Figure 6A**. The control group exhibited normal knee structures, with no signs of soft tissue edema or joint effusion. In contrast, the CIA group showed severe soft tissue edema, substantial joint effusion, and extensive trabecular and cortical bone destruction. Treatment groups displayed varying levels of improvement in these parameters. Notably, the FA-Cel-LPs group achieved the most notable reduction in soft tissue edema and preservation of bone integrity, further supporting the enhanced anti-RA efficacy associated with FA modification, possibly related to improved localization in inflamed joints. Compared to the CIA group, FA-Cel-LPs significantly increased BV/TV by ~80%, decreased BS/TV by ~45%, and restored BMD by ~60%, approaching levels in the healthy control group (**Figures 6B–D**). These results suggest that FA-Cel-LPs attenuated bone erosion and helped preserve trabecular microarchitecture in CIA rats.

### 3.12 MRI assessment of joint effusion and soft tissue damage

MRI images of rat left knee joints are presented in **Figure 6A**. The control group displayed normal knee joint structures, characterized by intact bone quality and normal joint spaces. In contrast, the CIA group exhibited pronounced structural abnormalities, including roughened surfaces of the medial and lateral condyles, tibial plateau, and patella, along with narrowed joint spaces and joint surface fusion, indicative of severe inflammatory damage. All treatment groups demonstrated varying degrees of improvement in attenuating bone erosion and maintaining structural integrity, with the FA-Cel-LPs group showing the most apparent improvement among the treatment groups.

### 3.13 Histopathological and immunohistochemical analysis of joint tissues

Histological images of H&E-stained knee joints from each group are shown in **Figure 6A**. The control group exhibited intact joint structures, normal joint spaces, no synovial hyperplasia, minimal infiltration of inflammatory cells, and no evident damage to cartilage or bone. In contrast, the CIA group displayed narrowed joint spaces, pronounced synovial cell proliferation (pannus formation), severe edema, extensive inflammatory cell and vascular infiltration, and severe cartilage and bone degradation. The treatment groups showed varying degrees of improvement, with reduced synovial hyperplasia and inflammatory cell infiltration, as well as alleviated cartilage and bone damage. Among them, the FA-Cel-LPs group demonstrated the greatest histological improvement and more closely resembled the control group than the other treated groups.

Immunohistochemical staining of knee joints (**Figure 6A**) revealed increased expression of the inflammatory markers COX-2 and iNOS in the CIA group, particularly in the cartilage and subchondral bone, compared to the control group. The treatment groups exhibited reduced expression levels of COX-2 and iNOS, with the FA-Cel-LPs group showing the most substantial reduction, approaching the baseline levels observed in the control group. These findings suggest that folate modification may improve the local anti-inflammatory efficacy of Cel, possibly by promoting greater accumulation of the formulation at inflamed sites.

### 3.14 Biosafety evaluation and systemic immunomodulation

**Figure 7:**
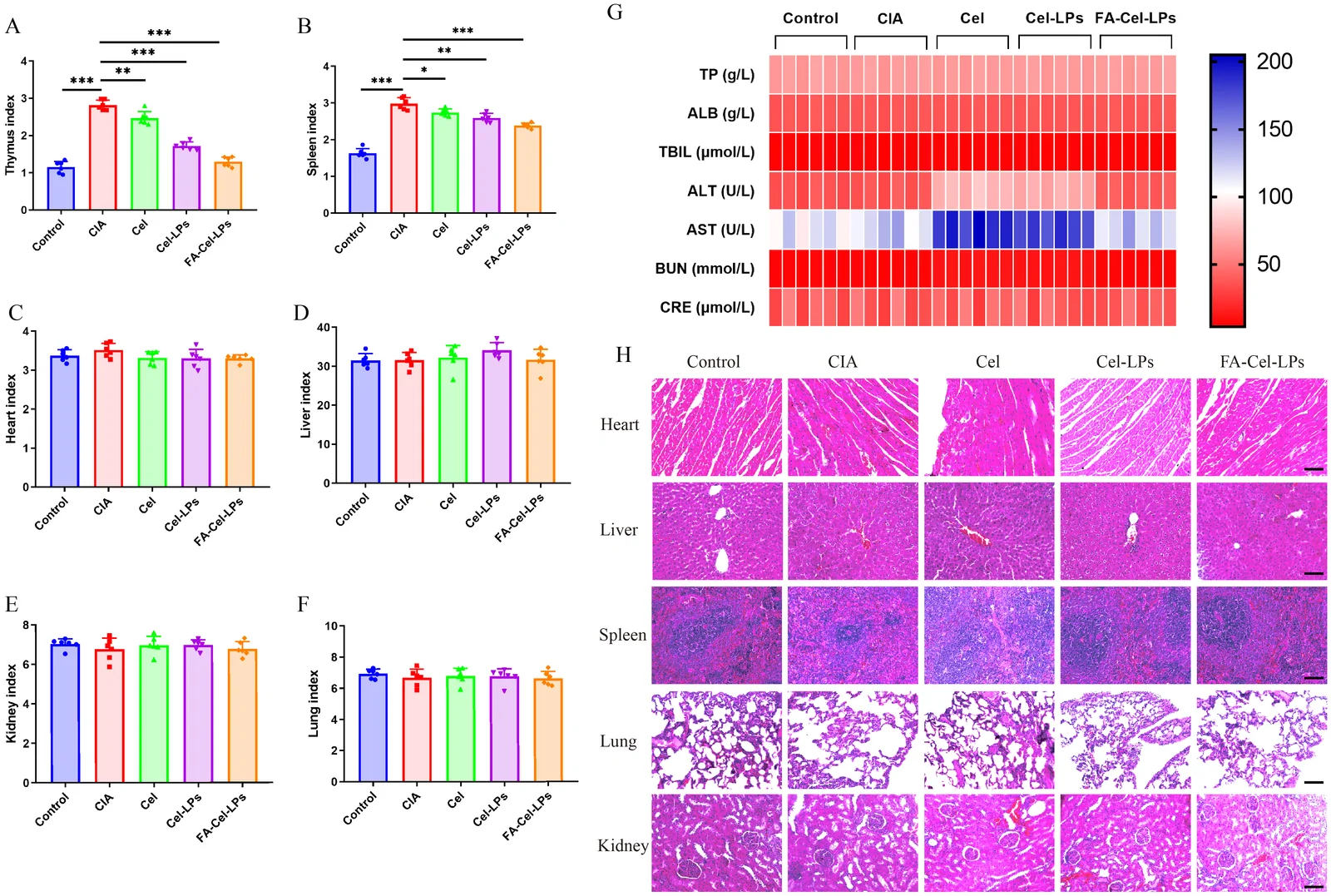
*In vivo* systemic biosafety evaluation of FA-Cel-LPs. **(A-F)** Relative organ indices of the thymus **(A)**, spleen **(B)**, heart **(C)**, liver **(D)**, kidneys **(E)**, and lungs **(F)** in CIA rats after different treatments (n = 6 per group). **(G)** Heatmap summarizing serum biochemical parameters in each group, including total protein (TP), albumin (ALB), total bilirubin (TBIL), aspartate aminotransferase (AST), alanine aminotransferase (ALT), blood urea nitrogen (BUN), and creatinine (CREA), to visualize overall differences in hepatic and renal safety profiles among groups. Higher and lower relative levels are represented by the corresponding color gradient in the heatmap. **(H)** H&E staining of major organs (heart, liver, spleen, lungs and kidneys) from each treatment group at the experimental endpoint to assess potential systemic toxicity (scale bar = 50μm). All results are shown as the mean ± SD. *p<0.05, **p<0.01, ***p<0.001 compared with the untreated CIA group.

The organ index results for rats in each group are presented in **Figures 7A–F**. Compared to the control group, the CIA group showed significantly increased thymus and spleen indices. In contrast, the treatment groups exhibited reductions in both immune organ indices, with the FA-Cel-LPs group showing the most notable decrease, suggesting a stronger immunomodulatory effect than the other treatments. No significant differences were observed in the indices for the heart, lungs, liver, or kidneys across the groups.

Serum biochemistry analysis further supported the safety profile of the formulations, and the overall trends are summarized in the heatmap shown in **Figure 7G**. The heatmap illustrates group-wise differences in TP, ALB, TBIL, AST, ALT, BUN, and CREA, showing that the FA-Cel-LPs group displayed a biochemical profile closer to that of the control group than the free Cel group. In particular, serum ALT (39.0 ± 2.6 U/L), AST (112.3 ± 7.0 U/L), BUN (5.5 ± 0.3 mmol/L), and creatinine (39.0 ± 6.1 μmol/L) remained within normal physiological ranges and were significantly lower than those in the free Cel group (ALT: 75.3 ± 4.9 U/L; AST: 189.3 ± 11.0 U/L, both *P* < 0.001), indicating that liposomal encapsulation with folate modification was associated with reduced hepatorenal toxicity relative to free Cel. In addition, TP, ALB, and TBIL remained within the normal range and did not differ significantly among groups.

Furthermore, H&E staining was performed on the major organs, including the heart, liver, spleen, lungs, and kidneys (**Figure 7H**). The CIA and free Cel groups showed disrupted spleen architecture, characterized by lymphocyte proliferation and inflammatory infiltration, whereas the other treatment groups did not exhibit obvious histopathological abnormalities in the examined organs. Together with the low hemolysis rates observed *in vitro*, these findings suggest that FA-Cel-LPs showed acceptable preliminary biosafety and reduced systemic toxicity under the present experimental conditions.

## 4 Discussion

Cel is widely recognized as one of the most promising natural bioactive agents for RA treatment, boasting potent anti-inflammatory, antioxidant, and immunomodulatory properties (34, 35). However, its clinical translation is severely impeded by inherent pharmacological defects, most notably extreme hydrophobicity, a short biological half-life, low systemic bioavailability, and dose-limiting hepatorenal toxicity. To overcome these translational barriers, the integration of advanced drug delivery systems with precision targeting strategies is imperative. Surface modification of liposomes with FA exploits the overexpression of folate receptor-beta (FR-β) on activated macrophages within the inflamed rheumatoid synovium, enabling receptor-mediated endocytosis while preserving the stealth properties of PEGylated liposomes (36, 37). In the present study, FA-Cel-LPs were successfully developed and evaluated. The results indicate that this targeted nanoplatform improved the solubility, formulation stability, and inflamed-joint accumulation of Cel, and was associated with anti-arthritic effects and modulation of ROS/NF-κB/COX-2-related inflammatory changes, while showing a favorable short-term safety profile under the current experimental conditions.

The rational design of nanocarriers fundamentally dictates their biological outcomes. The formulated FA-Cel-LPs exhibited an ideal monodisperse particle size of approximately 110 nm and an encapsulation efficiency exceeding 80%. This specific size range may favor accumulation in inflamed joints under conditions of increased vascular permeability, while also helping to reduce rapid clearance to some extent (38, 39). Furthermore, the negative surface charge conferred by PEGylation prevented particle aggregation and enhanced serum stability (40). Interestingly, FA-Cel-LPs demonstrated a pH-responsive biphasic release profile. The accelerated drug release kinetics observed at pH 6.5 align with the acidic microenvironment characteristic of RA synovial fluid, potentially promoting preferential payload release at inflammatory sites (41). Notably, incomplete release was observed within 144 h under both pH conditions. This may be attributed to the high hydrophobicity of Cel, which favors its retention in the liposomal bilayer, together with the known limitations of dialysis-based methods for poorly water-soluble drugs. Therefore, the observed release plateau likely reflects sustained drug retention rather than formulation failure. However, it should be acknowledged that intra-articular pH *in vivo* is dynamic, spatially heterogeneous, and may not consistently fall within the narrow acidic range mimicked *in vitro*. Direct measurement of intra-articular release kinetics remains technically challenging, and the pH-responsive behavior should therefore be regarded as a contributing-rather than exclusive-mechanism of joint-localized release. This possibility of joint-localized delivery is further supported by our *in vitro* uptake and *in vivo* imaging assays, which showed that FA modification enhanced intracellular internalization in LPS-activated RAW264.7 cells and increased localized accumulation in arthritic rat joints. Notably, while FA competition assays demonstrated significantly reduced uptake of FA-Cel-LPs in the presence of excess free folate, this finding suggests the involvement of a folate receptor-related uptake process rather than definitively establishing FR-β-specific endocytosis. Rigorous discrimination between FR-β-specific endocytosis and nonspecific phagocytosis would require FR-β knockout/knockdown models or anti-FR-β blocking antibodies, which were not employed in the current study and constitute an important direction for future mechanistic validation.

Pro-inflammatory cytokines, particularly TNF-α, IL-1β, and IL-6, orchestrate RA pathogenesis by driving synovial inflammation, cartilage degradation, and osteoclastogenesis (42–46). Blocking these mediators is a primary therapeutic strategy. In this study, our qRT-PCR and immunofluorescence results showed that FA-Cel-LPs significantly downregulated the expression of TNF-α, IL-1β, IL-6, and iNOS in LPS-activated macrophages. Notably, FA-Cel-LPs showed a greater inhibitory effect on these inflammatory markers compared to non-targeted Cel-LPs and free Cel. This advantage may be attributable, at least in part, to folate-mediated targeting and enhanced intracellular delivery, although nonspecific uptake by activated macrophages may also have contributed. This potential advantage of FA modification was also reflected in the *in vivo* therapeutic evaluation, particularly in imaging findings and bone microstructure-related parameters such as BV/TV and BMD.

The NF-κB signaling pathway is a master regulator of the inflammatory cascade in RA. Upon activation, it drives the transcription of downstream cytokines and angiogenic factors (e.g., MMPs and VEGF), creating a positive feedback loop that exacerbates synovial hyperplasia and bone erosion (47–51). Recent studies indicate that Cel ameliorates RA primarily by targeting this pathway (15). Consistent with this, densitometric quantification of our Western blot analysis confirmed that FA-Cel-LPs effectively suppressed LPS-induced NF-κB activation, evidenced by the reduction in p65 and IκBα phosphorylation. By efficiently delivering Cel to interrupt this critical signaling hub, FA-Cel-LPs may contribute to attenuation of NF-κB-driven inflammation.

Oxidative stress and local inflammation are intrinsically linked in RA pathology. Excessive ROS production exacerbates joint deterioration and amplifies inflammatory signaling (52, 53). Concurrently, upregulated iNOS and COX-2 promote the overproduction of nitric oxide and prostaglandin E2 (PGE_2_), accelerating pain and extracellular matrix degradation (54–56). Our *in vitro* and *in vivo* findings revealed that LPS-activated macrophages and CIA rat joints exhibit significantly elevated levels of ROS, iNOS, and COX-2. Treatment with FA-Cel-LPs reduced these oxidative and inflammatory markers, with a greater effect than free Cel. However, although these coordinated changes in ROS, NF-κB activation, COX-2, and iNOS are consistent with involvement of a redox-inflammation signaling axis, the present data remain correlative and do not establish a definitive causal dependence of therapeutic efficacy on the ROS/NF-κB/COX-2 pathway. Definitive causal validation will require future studies using specific NF-κB inhibitors (e.g., BAY 11-7082), ROS scavengers (e.g., NAC), or pathway-specific genetic knockdown.

These cellular and molecular findings were further supported by the therapeutic efficacy of FA-Cel-LPs in the CIA rat. While free Cel at a dose of 1 mg/kg produced a measurable therapeutic effect (11, 17), its efficacy is often constrained by poor bioavailability. Our results demonstrated that FA-Cel-LPs showed greater improvements than free Cel, resulting in a more profound reduction in paw swelling, arthritis score, and immune organ indices (spleen and thymus). Furthermore, high-resolution micro-CT, MRI, and histological analyses confirmed that FA-Cel-LPs alleviated joint effusion, pannus formation, and bone erosion. Quantitative micro-CT analysis of the knee joint provided objective evidence of bone microstructure preservation: FA-Cel-LPs increased BV/TV, reduced BS/TV, and restored BMD, approaching the values of healthy controls. These macroscopic and microscopic improvements highlight the synergistic therapeutic benefits of liposomal encapsulation and active joint targeting.

A major translational hurdle for nanomedicines and Cel is systemic toxicity (57). By increasing accumulation in inflamed joints, FA-Cel-LPs may help reduce off-target exposure relative to free Cel, although off-target distribution cannot be fully excluded. This was corroborated by H&E staining of major organs (heart, liver, spleen, lungs, and kidneys), which revealed no significant structural damage or inflammatory infiltration in the FA-Cel-LPs group. More importantly, serum biochemistry provided objective evidence supporting reduced systemic toxicity: serum ALT, AST, BUN, and creatinine in the FA-Cel-LPs group remained within physiological ranges and were significantly lower than in the free Cel group, indicating that folate-modified nano-encapsulation was associated with reduced hepatorenal toxicity relative to free Cel. Additionally, the hemolysis rate of our formulation was well below the internationally recognized 5% safety threshold (58). Although FR-β is upregulated on activated synovial macrophages, FR-α is also expressed in kidney proximal tubules, choroid plexus, and certain epithelial tissues, and FR-β may be expressed at low levels on some non-pathological macrophage populations. Off-target accumulation in these tissues, although likely limited by PEG stealth and inflammation-driven EPR localization, cannot be entirely excluded. The favorable serum biochemistry observed here provides indirect reassurance, but dedicated biodistribution and tissue-level FR-β co-localization studies are warranted in future work. Overall, the available data suggest that FA-Cel-LPs possess a favorable short-term safety profile for intravenous administration and represent an improvement over free Cel and Cel-LPs in this regard.

Despite these promising outcomes, this study has several limitations. First, the *in vivo* fluorescence imaging was qualitative rather than ROI-based quantitative biodistribution analysis and no *in vivo* pharmacokinetic profiling (Cmax, AUC, t_1/2_, or joint-tissue PK) was performed; pharmacokinetic claims were therefore reframed in terms of solubility, stability, and joint accumulation. Second, long-term toxicity, immunogenicity, and PEG-associated accelerated blood clearance were not assessed. Third, the anti-inflammatory effects were validated in RAW264.7 macrophages, but not in primary synovial macrophages derived from RA patients or animal models. Fourth, no standard-of-care positive control, such as methotrexate or dexamethasone, was included, which limits direct therapeutic benchmarking. Fifth, mechanistic involvement of the ROS/NF-κB/COX-2 axis was inferred from associated molecular changes but not formally validated using inhibitor-based or genetic approaches. Sixth, FR-β-specific uptake was not distinguished from nonspecific phagocytosis using genetic models. Finally, dose-response optimization of *in vivo* Cel dosing was not performed. These issues warrant further investigation to better define the translational potential of this formulation.

## 5 Conclusion

In conclusion, FA-Cel-LPs were developed as a folate-modified liposomal formulation for RA treatment. This formulation improved the solubility, stability, and inflamed-joint accumulation of Cel, and showed anti-inflammatory and bone-protective effects in experimental RA. These effects were associated with reduced oxidative stress and suppression of NF-κB/COX-2/iNOS-related inflammatory signaling. In CIA rats, FA-Cel-LPs alleviated joint inflammation and attenuated bone erosion, with no obvious short-term systemic toxicity under the present study conditions. Overall, these findings support further investigation of FA-Cel-LPs as a folate-modified Cel delivery strategy for RA.

## Data Availability

The original contributions presented in the study are included in the article/**Supplementary Material**. Further inquiries can be directed to the corresponding author/s.
