# Supplementary material for: Folate-modified liposomal celastrol for rheumatoid arthritis: therapeutic efficacy and modulation of ROS/NF-κB/COX-2-related inflammatory signaling
Source: Front Immunol. 2026 Sep 1;17:1841496. doi: 10.3389/fimmu.2026.1841496 (PMC13575751; doi:10.3389/fimmu.2026.1841496)
Supplement: Supplementary file 1 [file DataSheet1.pdf]

Supplementary Information

S1. UPLC-MS/MS analysis of celastrol

Celastrol concentrations for encapsulation efficiency (EE), drug-loading capacity (DLC), and in vitro release studies were quantified using an Acquity UPLC system coupled to a Xevo TQD triple quadrupole mass spectrometer equipped with an electrospray ionization source (Waters Corp., Milford, MA, USA). Chromatographic separation was achieved on an Acquity UPLC HSS T3 column (2.1 × 100 mm, 1.8 μm) maintained at 40°C. The mobile phase consisted of acetonitrile (solvent A) and 0.1% formic acid in water (solvent B), delivered at a flow rate of 0.4 mL/min. The gradient program was as follows: 0-0.5 min, 30% A; 0.5-1.0 min, 95% A; 1.0-2.0 min, 95% A; 2.0-2.3 min, 10% A.

Mass spectrometric detection was performed in positive electrospray ionization mode using multiple reaction monitoring (MRM). The source temperature was set at 150°C, and the desolvation temperature was 500°C. The cone gas flow and desolvation gas flow were 50 L/h and 1000 L/h, respectively. Celastrol and midazolam (used as the internal standard) were monitored using the transitions m/z 361.12 → 91.02 and m/z 325.98 → 291.07, respectively. The cone voltages were 58 V for celastrol and 50 V for midazolam, and the collision energies were 44 V and 26 V, respectively.

A celastrol stock solution (1 mg/mL) was prepared by dissolving 2 mg of celastrol powder in 2 mL methanol. The stock solution was further diluted with methanol to obtain a series of working solutions at concentrations of 5, 2.5, 1, 0.5, 0.25, 0.1, and 0.05 μg/mL. For calibration, 5 μL of each working solution was added to 150 μL acetonitrile containing midazolam (500 ng/mL). The mixture was vortexed thoroughly and centrifuged at 13,000 rpm for 5 min. Subsequently, 150 μL of the clear supernatant was transferred into an autosampler vial for UPLC-MS/MS analysis. Calibration curves were generated by plotting the peak area ratio of celastrol to the internal standard against celastrol concentration, followed by linear regression analysis.
